# Improving equity and access to buprenorphine treatment through telemedicine at syringe services programs

**DOI:** 10.1186/s13011-022-00483-1

**Published:** 2022-07-15

**Authors:** Barrot H. Lambdin, David Kan, Alex H. Kral

**Affiliations:** 1 RTI International, 2150 Shattuck Avenue, Suite 800, Berkeley, CA 94704 USA; 2 Bright Heart Health, Walnut Creek, CA USA

**Keywords:** Buprenorphine, Syringe service programs, People who inject drugs, Opioid treatment, Harm reduction

## Abstract

**Background and aims:**

In the United States, access to buprenorphine remains low and disparities regarding who receives treatment have emerged. Federal laws have regulated buprenorphine delivery, ultimately limiting its implementation more broadly. At the onset of the COVID-19 pandemic, federal agencies acted quickly to remove a legal barrier, effectively allowing people with opioid used disorder (OUD) to initiate buprenorphine treatment via telemedicine. Leveraging this policy shift, a low barrier buprenorphine treatment initiative via telemedicine was started at syringe service programs in California. We assessed early findings from participants reached by this model of treatment.

**Methods:**

In May 2020, buprenorphine treatment was offered through a virtual platform to SSP participants in California. SSP staff connected interested participants to virtual appointments with medical providers in a private location. During these visits, clinicians conducted clinical assessments for diagnosing participants with OUD and developed an unsupervised home induction plan for individuals who were eligible. Participants were prescribed a 7-day supply of up to 16 mg daily buprenorphine or 16 mg buprenorphine-2 mg naloxone and asked to return the following week if interested in continuing treatment.

**Results:**

From May 2020 to March 2021, the SSP-buprenorphine virtual care initiative inducted 115 participants onto treatment with 87% of participants inducted on the same day as their referral. Of those inducted, 58% were between the ages of 30 and 49 and 28% were cisgender female. Regarding participants’ method of payment to reimburse buprenorphine costs, 92% of participants were covered by Medicare/Medicaid. Overall, 64% of participants returned for a second buprenorphine prescription refill.

**Conclusions:**

These early findings suggest that this could be a promising approach to improve equity and access to buprenorphine treatment. We encourage policymakers to continue allowing buprenorphine delivery via telemedicine and researchers to study whether this approach improves equity and access to treatment throughout the United States.

## Background

An estimated 1.6 million people have an opioid use disorder (OUD) in the United States (US), with merely 18% of people receiving medications for their OUD (MOUD) [1]. Buprenorphine, a MOUD approved by the US Food and Drug Administration in 2002, has a strong evidence-base showing reductions in opioid use and opioid overdose mortality [2, 3]. Of the approved MOUDs, buprenorphine is considered to have the greatest opportunity for expanded access in the US, because it does not require patients to visit specialty clinics on a daily basis [2, 4]. However, major barriers exist in ensuring access, especially to our most vulnerable communities [5].

Federal laws have limited buprenorphine implementation in the US more broadly [6]. Specifically, the Ryan Haight Act of 2008 requires that an initial prescription of a controlled substance like buprenorphine involves an in-person examination between a potential patient and a trained provider [6]. In addition to the Ryan Haight Act, the majority of highest need counties in the US have an inadequate number of trained providers [2], and when trained providers do exist, many have not prescribed buprenorphine or do so well below their capacity because they have few to no patients with OUD [2], insufficient time to prescribe or blame poor reimbursement rates [7]. As a result, access to buprenorphine is limited, especially so among vulnerable communities. Recent studies have shown that buprenorphine treatment access was concentrated among people who have private insurance, can pay out of pocket, and are white [8]. Identifying new delivery models that that can bridge the gap between demand for and access to MOUD is critically important to reduce disparities in buprenorphine access and ensure benefits of this life-saving medication are extended equitably.

Syringe service programs (SSPs) provide access to and disposal of sterile syringes and injection equipment for people who use drugs and have been a linchpin for public health efforts to prevent HIV, HCV, and overdose fatalities among people who use drugs [9]. An estimated 431 SSPs are operating in the US [10]. SSPs are ideal venues to consider for buprenorphine delivery; SSP services are offered in community-based settings that are easily accessible to participants; SSP staff are culturally competent in providing services with dignity and respect. SSP participants have a higher likelihood of opioid overdose and OUD, trust these organizations to care for their health and are less likely to access care in other healthcare settings [11]. In addition, SSPs generally serve people without private insurance and without the ability to pay out-of-pocket [12–14]. As such, SSPs are an ideal venue for connecting interested and eligible people who have previously faced limited access to buprenorphine treatment. Recent initiatives have illustrated the emergence of low-barrier buprenorphine treatment for marginalized populations, including descriptions of telehealth buprenorphine models at SSPs [15–18]. Research has shown that co-locating in-person buprenorphine treatment at SSPs can increase access and achieve similar levels of treatment outcomes to those observed in primary care and specialty settings [19, 20].

One factor that has limited large-scale adoption of buprenorphine at SSPs is that it required clinicians to be on-site when participants present for syringe services. With limited geographic coverage of trained providers, this would be a major impediment for the majority of SSPs. Furthermore, many SSPs in rural settings that operate primarily through outreach and mobile services face additional barriers to including clinicians within their service structure.

At the beginning of the COVID-19 pandemic, the US Drug Enforcement Agency, in concert with other federal agencies, took unprecedented measures to remove some legal barriers for buprenorphine [6]. In particular, the Ryan Haight Act’s requirement for an in-person examination to prescribe buprenorphine was waived. This waiver has allowed medical providers to prescribe buprenorphine and induct patients through virtual platforms, facilitating a streamlined connection between trained medical providers and people with OUD. Leveraging this opportunity, we implemented a telemedicine initiative for buprenorphine inductions at SSPs in California and assessed whether the approach could improve access to this essential medication.

## Methods

In May 2020, Bright Heart Health began offering low threshold buprenorphine or buprenorphine-naloxone access through a virtual platform to participants at SSPs in California. SSP participants were offered appointments with medical providers via two-way video connections during the SSPs’ operating hours, with tablets provided by the SSP. Four SSPs began referring participants – 1 implemented telemedicine buprenorphine in May 2020, a second implemented in July 2020 and two more implemented in December 2020.

SSPs informed participants as they arrived for services that telemedicine buprenorphine was now offered through the SSP, and interested participants were connected to staff who were trained substance use navigators. These navigators initially conducted brief assessments to establish an OUD diagnosis; then, provided education and determined interest in MOUD. For interested participants, SSP staff described and answered questions about the program and then connected participants to real-time virtual appointments with Bright Heart medical providers in a private location at the SSP. These staff maintained ongoing contact with participants, helping with prescription fills and refills on a case-by-case basis. Though not a requirement, these navigators often had lived experience with drug use and medications for OUD.

During virtual visits, clinicians acquired consent from individuals, conducted clinical assessments for diagnosing participants with OUD and developed an unsupervised home induction plan for individuals who were eligible. To facilitate access to treatment with as few barriers as possible, data collection was minimal yet consistently included age, gender and payment type – Medicaid, Medicare, private insurance or self-pay. Participants who were inducted were prescribed a 7-day supply of up to 16 mg daily buprenorphine or 16 mg buprenorphine-2 mg naloxone and asked to return the following week if they were interested in continuing with treatment. Follow-up appointments could occur at the SSP or wherever the participant was located. Participants received their medication each week from local pharmacies in collaboration with the SSP and were offered other social and behavioral health support services when appropriate. Prescription fills and refills were verified by authorized providers through California’s Controlled Substance Utilization Review and Evaluation System – a database that monitors all Schedule II-IV controlled substances dispensed to people in California. No urine drug screening was required. Participants could continue with Bright Heart health providers indefinitely, or they could transfer to a different program if they preferred. With regards to data used in this study, information from each visit was entered into Bright Heart Health’s electronic medical record.

 Our study procedures were reviewed by an internal review board at Ethical and Independent Review services, which determined that these study procedures met requirements for exemption from IRB review.

## Results

 From May 28, 2020 to March 26, 2021, the SSP-buprenorphine virtual care initiative inducted 114 participants onto treatment. Of those inducted, 58% were between the ages of 30 and 49 and 28% were cisgender female (see Table 1). Overall, 87% of participants were inducted on the same day as their referral, with 6% inducted 1 day after their referral and 7% inducted more than 1 day after their referral. Regarding participants’ method of payment to reimburse buprenorphine treatment costs, 92% of participants were covered by Medicaid or Medicare, 4% of participants were covered by private insurance, and 4% of participants were covered through self-pay. Overall, 64% of participants returned for a second buprenorphine prescription fill.

**Table 1:** Characteristics of SSP participants inducted onto buprenorphine (*N* = 115)

	n (%)
Age (in years)	
<30	17 (15%)
30–39	35 (30%)
40–49	32 (28%)
≥50	31 (27%)
Gender	
Cisgender male	83 (72%)
Cisgender female	32 (28%)
Time to Induction	
Same Day as Referral	100 (87)
One Day after Referral	7 (6)
More than One Day after Referral	8 (7)
Payment method	
Medicaid/Medicare	106 (92%)
Private	5 (4%)
Self-pay	4 (4%)
Prescription refill	74 (64%)

## Discussion

To effectively address OUD and the rising levels of opioid-related overdose mortality in the US, we must improve access to and uptake of MOUD like buprenorphine. A key element of this work will be to address and reduce disparities in buprenorphine treatment access for vulnerable populations. At the onset of the COVID-19 pandemic, the waiver of in-person examination requirements to receive buprenorphine opened the possibility for new innovations for providing treatment to people with an OUD. As a result, the use of virtual platforms to conduct medical appointments for buprenorphine treatment at SSPs effectively connected medical providers to new settings and new participants who had a need and demand for treatment. In the first ten months of operation, our data showed that 92% of PWUD who initiated buprenorphine were covered by Medicare or Medicaid. As public insurance programs, Medicaid and Medicare provide health coverage to communities typically not included in private insurance – people with low income, seniors and/or people with disabilities. Therefore, this model can potentially expand buprenorphine treatment access to lower income and more vulnerable populations who have limited access to this treatment relative to people with private insurance.

Furthermore, 64% of participants inducted onto buprenorphine returned for a refill. While more research is needed with this approach, a recent study from Canada demonstrated that patients receiving MOUD via telemedicine were 26% more likely to be retained in care for 12 months, compared to those receiving in-person MOUD [21]. Other studies have shown that a similar approach led to improvements in patient satisfaction and reductions in illicit drug use [22, 23]. Offering participants access to telemedicine-based buprenorphine through the SSP removed important barriers to buprenorphine access. First, the approach addressed geographic and resource barriers - participants did not have to spend time and money travelling to a different location to meet with a prescribing clinician; and prescribing clinicians did not need to travel to SSPs to meet with interested participants. Relatedly, this model allows clinicians to serve participants from multiple SSPs on the same day, thereby optimizing use of the limited number of trained providers. In addition, embedding telemedicine-based buprenorphine within SSPs might be a better approach for reaching participants who avoid other health care settings due to experiences of discrimination within them [24]. With ten months of data, a limitation of our study is that we did not have sufficient data or number of participants to assess uptake and retention of participants over time.

## Public health implications

While more research is needed across a broader array of SSPs in the US, our findings suggest that this could be a promising approach that can address the gap between demand for and access to buprenorphine treatment. By reaching a segment of the population with historically limited treatment access, SSP-based telemedicine buprenorphine could be a critical component to improve equity and access to buprenorphine treatment among people with an OUD.

## Data Availability

The datasets used and/or analyzed during the current study are available from the corresponding author on reasonable request.
